# Development and validation of Age-Specific algorithms for diabetes prediction

**DOI:** 10.1007/s12020-025-04428-z

**Published:** 2025-09-23

**Authors:** Shigehiro Karashima, Haruka Nishida, Yu Ishikawa, Ren Mizoguchi, Atsushi Hashimoto, Toshitaka Sawamura, Akihiro Nomura, Hayato Tada, Kenji Furukawa, Akitaka Higashi, Hiroyuki Mori, Kohei Hirako, Yuma Morisaki, Makoto Fujiu, Hidetaka Nambo

**Affiliations:** 1 Institute of Liberal Arts and Science, Kanazawa University, Kanazawa, Japan; 2 School of Electrical Information Communication Engineering, College of Science and Engineering, Kanazawa University, Kanazawa, Japan; 3 College of Transdisciplinary Sciences for Innovation, School of Entrepreneurial and Innovation Studies, Kanazawa University, Kanazawa, Japan; 4 Department of Health Promotion and Medicine of the Future, Kanazawa University Graduate School of Medicine, Kanazawa, Japan; 5 Faculty of Transdisciplinary Sciences, Institute of Philosophy in Interdisciplinary Sciences, Kanazawa University, Kakuma-machi, Kanazawa, 920-1192 Japan; 6 Department of Internal Medicine, Asanogawa General Hospital, Kanazawa, Japan; 7 Department of Cardiovascular Medicine, Kanazawa University Graduate School of Medical Sciences, Kanazawa, Japan; 8 Health Care Center, Japan Advanced Institute of Science and Technology, Nomi, Japan; 9 Emerging Media Initiative, Kanazawa University, Kanazawa, Japan; 10 Department of Molecular & Integrative Physiology, University of Michigan Medical School, Ann Arbor, MI USA; 11 The Faculty of Interdisciplinary Economics, Kinjo University, Ishikawa, Japan

**Keywords:** Machine learning, Age-specific models, Light gradient boosting machine, Anomaly detection

## Abstract

Diabetes mellitus (DM) has a higher incidence among older adults. This study aimed to develop age-specific DM prediction models using machine learning (ML) and anomaly detection algorithms. We included 489,073 participants from Kanazawa City and 31,923 from Hakui City who underwent health check-ups. Four models were constructed, comprising a Light Gradient Boosting Machine (LGBM), TabNet, Variational Autoencoder, and Isolation Forest (IF), to predict DM onset within three years. The models were trained using the Kanazawa dataset and externally validated using the Hakui dataset. Performance was evaluated based on the area under the curve (AUC), sensitivity, and specificity. The LGBM model demonstrated the highest AUC across multiple age groups in both internal and external validations. For participants in their 50s and 60s, the LGBM achieved AUC values of 0.911 during internal validation, with sensitivity and specificity exceeding those of the other models. In contrast, the IF model exhibited the best performance for participants in their 40s. The findings of this study suggest the potential effectiveness of age-specific models in improving diabetes prediction accuracy within the study population. Further validation using more diverse populations and younger age groups are recommended for future research.

**Supplementary Information:**

The online version contains supplementary material available at 10.1007/s12020-025-04428-z.

## Introduction

Diabetes mellitus (DM) is a severe public health challenge, generating an enormous social and economic burden [1]. It is a prevalent lifestyle disease, with more than 10% of the global population estimated to be have DM or be at a high risk of developing it [1]. Its prevalence varies by generation, with older age groups having higher rates of diabetes [1, 2]. It is the leading cause of cardiovascular disease, kidney failure, and blindness. Early therapeutic intervention for diabetes is crucial in mitigating long-term complications and reducing mortality rates. Recent research by Lind et al. demonstrated that patients who achieved a 1% reduction in glycated hemoglobin (HbA1c) within the first year of diagnosis exhibited significantly lower mortality and myocardial infarction rates than those who achieved a similar reduction in blood glucose levels over the subsequent 5–10 years [3]. Therefore, early diagnosis and prompt intervention are essential. Nevertheless, practical challenges often result in a delay between elevation in blood glucose levels and a formal diagnosis of DM [4, 5]. It is, therefore, important to predict which individuals are at a high risk of developing DM, even before its onset.

Recently, several algorithms for predicting diabetes onset using big data from health checkups have been reported [6–8], most of which use machine learning (ML) models. Zou et al. reported the prediction of new-onset diabetes from the data of approximately 150,000 inpatients, and a random forest model showed the highest prediction accuracy, with an area under the receiver operating characteristic curve (AUC) of 0.8084 [7]. Zhang et al. used data from 36,652 participants in a cohort study using gradient boosting, achieving an AUC of 0.872 for new-onset DM [8]. Hence, ML may be a promising tool for improving prediction performance compared to traditional statistical prediction models [9]. It may, however, be difficult to develop useful models, because an imbalance in the number of cases between the disease and non-disease groups may cause one to overlearn the other.

Furthermore, Fregoso-Aparicio et al. noted that diabetes predominantly affects older adults, leading to possible age-related imbalances in datasets and potentially affecting model accuracy [10].

Therefore, in this study, we focused on optimizing prediction models by age group to address the variations in diabetes prevalence across generations, with the aim of improving the accuracy of predictions within each group. The development of age-specific models may allow for better performance than a general model that uses all-age data. Models that use deep learning for abnormality detection, such as the variational autoencoder (VAE) [11] and isolation forest (IF) [12], are particularly effective in handling imbalanced datasets where the majority of cases are non-diseased. These methods can mitigate the issue of overfitting, which is common in traditional ML models when disease prevalence is low [13]. For age groups with a low prevalence of DM, it may be possible to predict the disease with higher accuracy by changing the model using abnormality detection, instead of by using a conventional ML model to analyze age as a feature.

In this study, using the same database, we developed four models, including a light gradient boosting machine (LGBM) [14], TabNet [15], VAE [11], and IF [12], to predict the onset of DM within three years. By comparing prediction accuracies across different age groups, we aimed to demonstrate that age-specific models could outperform general models, thus providing more reliable predictions for each generation.

## Materials and methods

### Study participants

This study is a cross-sectional, observational study focusing on participants aged 40 years and older who underwent community-based health checkups conducted in Kanazawa City and Hakui City, Ishikawa Prefecture, Japan [16–18]. All clinicians received a manual detailing guidelines of each academic society and checkups were performed accordingly. During the checkups, they conducted standard medical examinations and recorded data such as height, weight, waist circumference, blood pressure, the results of biochemical examinations, and urinalysis. In addition, the health checkup participants filled out mark sheets and completed questionnaires regarding their disease history, lifestyle habits, and medications for hypertension, DM, and dyslipidemia. Medical information was obtained from clinics where health checkups were conducted and was centrally collected by the Kanazawa Medical Association. The exclusion criteria for the study were defined as residents already diagnosed or being treated for DM. The detailed requirements are as follows: (1) HbA1c levels ≥ 6.5%; (2) fasting blood glucose levels ≥ 126 mg/dL; (3) a self-reported history of diabetes; (4) use of oral hypoglycemic agents, insulin, or glucagon-like peptide 1 agonists.

The algorithm was developed and internally validated using data from 175,803 individuals who underwent health checkups in Kanazawa City between 2008 and 2018. External validation was performed using data from 17,575 individuals who underwent health checkups in Hakui City between 2012 and 2022.

## Ethical considerations

The study was conducted in accordance with the Declaration of Helsinki and Ethical Guidelines for Medical Research. Internal validation was approved by the Ethics Committees of the Kanazawa Medical Association and Kanazawa University (approval numbers: 16000003 and 2019-080, respectively). For external validation, a comprehensive partnership agreement was established between Kanazawa University and Hakui City in 2017 that included the use of specific health checkup data. The use of these data for external validation was reviewed and approved by the Ethics Committee of the Division of Transdisciplinary Sciences at Kanazawa University (approval number YUR4-003). Due to the secondary nature of the data, informed consent was not required. The study details were published on the websites of Kanazawa University, Kanazawa Medical Association, and Hakui City, providing an opt-out option for participants.

## Features

The health checkup database includes a range of clinical parameters including physical observations and medical histories. Basic characteristics such as age, sex, body mass index (BMI), waist circumference, systolic blood pressure, and diastolic blood pressure, as well as physical findings such as jaundice, arrhythmia, heart murmur, crackles, hepatomegaly, edema, cervical tumor, neuropathy, malnutrition, and anemia were collected from the dataset.

Furthermore, the following blood data were measured, including white and red blood cell counts; hemoglobin levels; hematocrit; mean corpuscular volume (MCV); mean corpuscular hemoglobin (MCH); mean corpuscular hemoglobin concentration (MCHC); platelet count; serum creatinine levels; estimated glomerular filtration rate; and levels of serum uric acid (UA), aspartate aminotransferase, alanine transaminase, γ-glutamyl transpeptidase, plasma glucose, HbA1c, total cholesterol (TC), triglyceride, low-density lipoprotein-cholesterol, and high-density lipoprotein-cholesterol (HDL-C). Blood samples were analyzed within 24 h of collection using an automated clinical chemistry analyzer, in accordance with specimen testing methods recommended by the Japanese Society for Clinical Chemistry. Urinary protein, glucose, and occult blood were examined semi-quantitatively in five levels: “negative,” “trace,” “1+,” “2+,” and “3+.

In addition, electrocardiographic findings were evaluated in ten categories (normal, abnormal Q wave, QRS axis deviation, R-wave elevation, ST depression, T-wave abnormality, atrioventricular conduction defect, ventricular conduction defect, arrhythmia, and other abnormalities) according to the Minnesota codes. The mark-sheet questionnaire contained the following: drug history (hypertension, diabetes, and dyslipidemia); history of 4 diseases (stroke, coronary artery disease, chronic kidney disease, or anemia); and 13 items related to lifestyle: (1) smoking, (2) weight gain (> 10 kg/20 years), (3) exercise (> 30 min, twice a week, > 1 year), (4) daily walking or equivalent (> 1 h), (5) walking faster (than others in the same generation), (6) body weight changes (> 3 kg/year), (7) eating faster (than others in the same generation), (8) eating within 2 h before going to bed (> 3/week), (9) having a snack after dinner (> 3/week), (10) skipping breakfast (> 3/week), 11) daily drinking (alcohol), 12) heavy drinking (> 60 g ethanol/day), and 13) good sleeping, by themselves [16–18] (Supplemental Table S1).

## Dataset construction

The dataset for predicting DM onset within three years was constructed using the following procedure. First, the year in which the subject first underwent a medical checkup was identified, and data from that year were used as explanatory variables. Next, data from medical checkups conducted within three years of the base year were used to set the outcome variable. With regard to the potential outcome variables, if multiple years of data were available within a three-year period, only the results from the earliest year were selected. This process was repeated to collect the data. After data collection, subjects who developed DM by the base year were excluded, and the final dataset was constructed.

## Statistical analysis for clinical background

Data are expressed as means ± standard deviations or percentages. The Shapiro–Wilk test was used to assess normality. For data that were not normally distributed, the Mann–Whitney *U* test was used. If the distribution was normal, Bartlett’s test was used for equal variance, and the Student’s *t*-test or Welch’s t-test was used if equivariance was found or absent, respectively. A *P* < 0.05 indicated statistical significance. Python 3.8.3 programming language (Python Software Foundation, Delaware, USA) and SciPy 1.5.2 were used for statistical analysis.

### Model construction

In this study, we constructed four predictive models: Light Gradient Boosting Machine (LGBM), TabNet, Variational Autoencoder (VAE), and Isolation Forest (IF). The Light Gradient Boosting Machine (LGBM) is an efficient gradient-boosting framework based on decision trees, designed for high-speed computation and high predictive accuracy, even with large-scale datasets. TabNet is an attention-based deep learning architecture optimized for tabular (structured) data, which uses sequential attention to select the most relevant features at each decision step, enabling both high accuracy and interpretability. The Variational Autoencoder (VAE) is a neural network model used for anomaly detection, which learns the normal patterns of the data and identifies unusual cases based on reconstruction errors. The Isolation Forest (IF) is an anomaly detection algorithm that isolates observations by randomly selecting features and split values, making it particularly effective for detecting rare cases in highly imbalanced datasets.

Models were constructed using the Python software. Imbalanced-learn [19], Scikit-learn [20], lightgbm [21], pytorch_tabnet [22], and Tensorflow [23] were used for undersampling and to build the IF, LGBM, TabNet, and Variational Autoencoder (VAE) models, respectively.

## Handling of missing data

In this study, missing values were handled using a consistent approach across models. For Light Gradient Boosting Machine (LGBM), missing values were processed internally by the algorithm, whereas for all other models (TabNet, VAE, and Isolation Forest), missing values were imputed prior to model construction using the MissForest algorithm [24].

## Feature selection process

Feature selection was conducted using all available clinical and lifestyle variables from the health check-up dataset as initial inputs. Model-specific feature importance scores—Gini importance for Random Forest, gain-based importance for LGBM, and attention-based feature masks for TabNet—were calculated on the training data. Features were ranked according to these scores, and the subset that achieved the highest mean AUC across 50 repeated trials was adopted for final modeling. LGBM and TabNet selected 10 features, whereas the anomaly detection methods VAE and IF did not perform feature selection.

### Hyper parameters for TabNet and VAE

For the TabNet, the implementation of TabnetClassifier on PyTorch is applied. The hyper parameters are used as default except evaluation metric (eval_metric). In this experiment, AUC is used for evaluation metric (default is Accuracy). For default values, please see the official documents [22]. For the VAE, we implemented the VAE network with tensorflow keras framework. The network is consisted from 2 parts, the encoder and the decoder. The encoder consists of 2 dense layers and after that, average and log variance are calculated for the latent space. Then, from the latent, the average and the variance, the decoder consisted of 2 dense layers decodes and reconstruct the output. Here, the size of the first layer of the encoder and the last layer of decoder, these are an input layer and an output layer, are same as the size of input data. The size of the second layer of the encoder and the first layer of the decode are half of the size of input data. Adam is used for optimizer, epochs is 30 and batch size is 32. The latent dimension is also half of the size of input data, and standard Gaussian prior over the latent space. Other parameters are default value. Please see the official documents [25].

### Procedure of classification models (LGBM and TabNet)

For TabNet, missing values were imputed using MissForest [25], whereas missing value handling was not performed for the LGBM model. The database was divided into two distinct datasets: the training set comprised data collected from 2008 to 2018, and the dataset was split into training (80%) and test (20%) data. Stratified sampling was used to ensure that the ratio of diabetic to non-diabetic patients in the training and test datasets was consistent with the presplit ratio.

Subsequently, under-sampling was performed to address the class imbalance. Parameter tuning and feature selection were conducted using the training set, and model performance was evaluated based on an average of 50 replicates. A feature set that maximized the AUC was selected. The feature selection for LGBM and TabNet was determined based on the importance rankings derived from the training dataset for each model.

### Procedure of anomaly detection models (VAE and IF)

Missing values were imputed using MissForest and normalization was subsequently performed for the VAE. The dataset was split into training (80%) and test (20%) data, and stratified sampling was used to ensure that the ratio of diabetic to non-diabetic cases was equivalent in both the training and test datasets.

For the VAE, training was conducted using only data from non-diabetic individuals extracted from the training data. This subset was used to build and train ML models. Feature selection for IF utilizes the importance of isolated forest features [22]. The model performance was evaluated using test data and assessed based on an average of 50 replicates. The threshold for the VAE was determined using the Youden index [26].

### Performance evaluation

The generalization performance of the model developed using Kanazawa City data was evaluated in terms of the AUC, sensitivity, and specificity. Finally, the generalization performance was assessed using external validation with data from Hakui City.

## RESULTS

### Subject characteristics

**Fig. 1:**
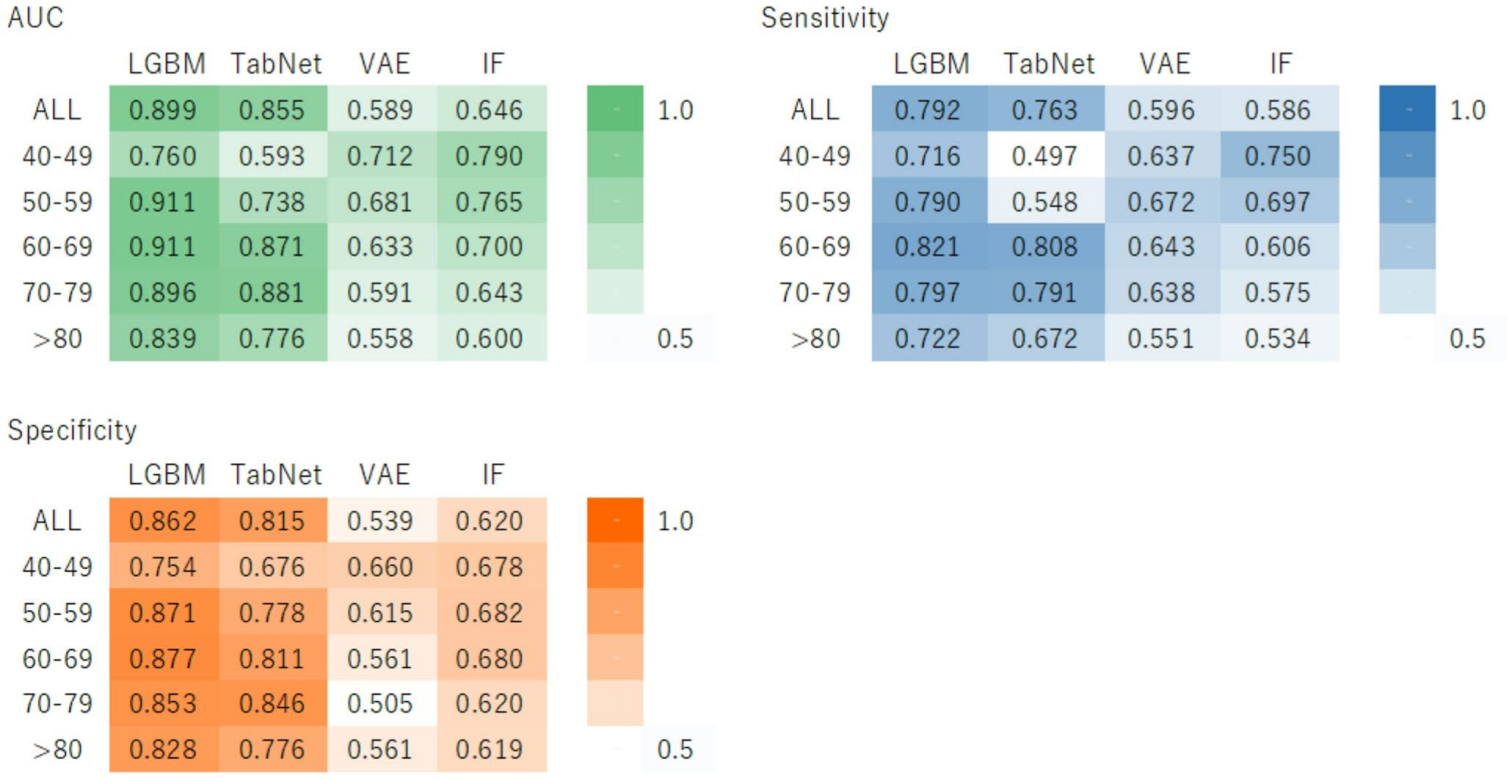
Prediction accuracy in internal validation presented as a heatmap. Panels A, B, and C show the area under the receiver operating characteristic curve (AUC), sensitivity, and specificity, respectively. Values represent the mean results from 50 independent internal validation trials. Models include Light Gradient Boosting Machine (LGBM), TabNet, Variational Autoencoder (VAE), and Isolation Forest (IF). Age groups are labeled directly on the heatmap as 40–49 years (40s), 50–59 years (50s), 60–69 years (60s), 70–79 years (70s), and ≥ 80 years (80s and older). In the heatmap, green indicates AUC, blue indicates sensitivity, and orange indicates specificity; darker shades indicate higher values for the respective metric

A total of 489,073 residents from Kanazawa City who underwent health checkups were enrolled for internal validation, while 31,923 residents from Hakui City were enrolled for external validation. Table 1 outlines the baseline characteristics of residents who underwent health checkups, categorized by age group. Using these data, we constructed an internal validation dataset comprising 175,804 cases and an external validation dataset comprising 17,575 cases. In the internal validation dataset, 4,213 patients developed DM within three years, whereas 171,591 did not. In the external validation dataset, 658 patients developed DM, whereas 16,917 did not. Figure 1 shows the proportion of participants who developed DM within three years. In Kanazawa City, the prevalence of DM was 0.62% for individuals in their 40s; 1.66% for those in their 50s; 1.98% for those in their 60s; 2.84% for those in their 70s; and 2.77% for those aged 80 years or older. In Hakui City, the prevalence was 0.86% among in individuals in their 40s; 4.33% for those in their 50s; 4.26% for those in their 60s; 3.85% for those in their 70s; and 2.70% for those aged 80 years or older.

**Table 1:** Baseline characteristics by ages

Kanazawa City	ALL	40–49	50–59	60–69	70–79	> 80
N	175,803	4981	10,494	58,300	67,493	33,690
Age (years)	72.1 ± 7.9	45.8 ± 2.8	56.9 ± 2.7	66.4 ± 2.7	75.3 ± 2.8	85.2 ± 3.7
Female (n, %)	113,157, (64.4)	3103, (62.3)	7061, (67.3)	37,296, (64.0)	42,290, (62.7)	22,861, (67.9)
BMI (kg/m^2^)	22.7 ± 3.2	22.5 ± 3.8	22.7 ± 3.6	22.7 ± 3.1	22.9 ± 3.1	22.50 ± 3.3
Waist circumference (cm)	83.2 ± 9.3	80.5 ± 10.3	81.9 ± 10.0	82.6 ± 9.0	84.0 ± 9.1	83.5 ± 9.7
FPG (mg/dL)	98.6 ± 19.4	91.7 ± 15.7	94.1 ± 16.2	95.2 ± 15.6	100.2 ± 20.6	103.7 ± 22.6
HbA1c (%)	5.4 ± 0.5	5.2 ± 0.4	5.3 ± 0.4	5.4 ± 0.4	5.5 ± 0.4	5.5 ± 0.4
Diabetes onset in 3yrs (n, (%))	4213, (2.40)	31, (0.62)	174, (1.66)	1156, (1.98)	1915, (2.84)	933, (2.77)
Hakui City	ALL	40–49	50–59	60–69	70–79	> 80
N	17,575	699	1225	7046	3921	2521
Age (years)	69.7 ± 10.2	45.5 ± 2.8	56.7 ± 2.9	66.2 ± 2.6	75.1 ± 2.9	85.2 ± 4.0
Female (n, %)	5859, (37.8)	364, (52.1)	456, (37.2)	2796, (39.7)	2796, (38.8)	1520, (27.0)
BMI (kg/m^2^)	22.9 ± 3.3	23.2 ± 4.0	23.3 ± 3.9	23.1 ± 3.4	23.2 ± 3.3	22.7 ± 3.5
Waist circumference (cm)	83.4 ± 9.1	82.8 ± 10.5	84.5 ± 11.0	83.9 ± 9.2	84.3 ± 9.1	84.2 ± 10.2
FPG (mg/dL)	94.5 ± 17.5	90.4 ± 12.5	94.4 ± 14.4	97.8 ± 21.6	101.5 ± 23.0	112.6 ± 30.9
HbA1c (%)	5.5 ± 0.6	5.5 ± 0.5	5.7 ± 0.7	5.8 ± 0.6	5.8 ± 0.6	5.7 ± 0.6
Diabetes onset in 3yrs (n, %)	658, (3.74)	6, (0.86)	53, (4.33)	300, (4.26)	151, (3.85)	68, (2.70)

The baseline clinical characteristics of collected subjects. The basic clinical values of participants from the health screenings in Kanazawa City and Hakui City are presented as the mean and standard deviation (SD). Median values are shown along with the 25th and 75th percentiles (1st quartile and 3rd quartile)

### Performance of the predictive model in internal validation

Figure 1 presents a heatmap of the AUC, sensitivity, and specificity for all age groups and for each specific age group for internal validation. The LGBM model demonstrated the highest AUC, sensitivity, and specificity across the datasets for all age groups. In terms of the AUC and sensitivity, the IF model exhibited the highest performance for individuals in their 40s, whereas the LGBM model showed the best performance for those in their 50s, 60s, 70s, and 80s.

For the age group of 50–59 years, the LGBM model achieved an AUC of 0.911 ± 0.030, sensitivity of 0.790 ± 0.088, and specificity of 0.871 ± 0.034, whereas in the 60–69 age group, it achieved an AUC of 0.911 ± 0.011, sensitivity of 0.821 ± 0.027, and specificity of 0.877 ± 0.007.

### Feature importance analysis in all age model

In the feature selection process, all candidate variables from the health check-up dataset were initially included. Model-specific feature importance measures were then calculated using the training dataset, and variables were ranked accordingly. For the all-age LightGBM model, the top 10 features (e.g., HbA1c, plasma glucose, medication for hypertension, estimated glomerular filtration rate, HDL-C, height, chewing, BMI, UA, MCH) were selected based on their contribution to the model’s predictive performance. For the all-age TabNet model, the top 10 features were selected, with plasma glucose and HbA1c again ranking highest, followed by gender, TC, platelet count, BMI, hematocrit, history of CKD, history of arrhythmia, and MCHC. The complete lists of feature ranking for both models are presented in Supplemental Figure S1.

### Performance comparison of the all-age model and age-specific models through external validation

**Fig. 2:**
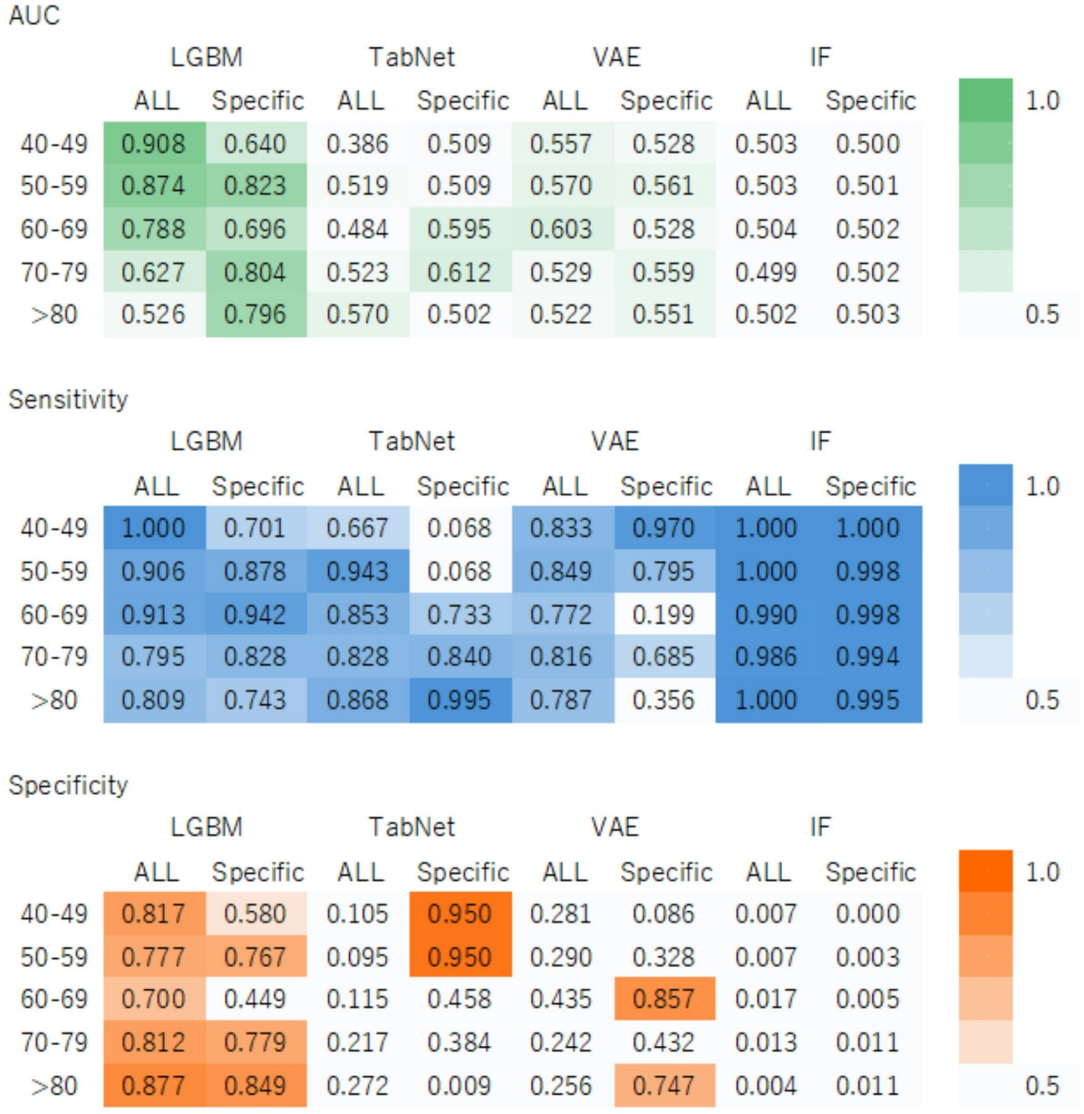
Comparison of external validation between the all-age model and the age-specific models. Panels A, B, and C use heatmaps to present the area under the receiver operating characteristic curve (AUC), sensitivity, and specificity, respectively. Values in each cell represent the results obtained from a single external validation trial using the full external validation dataset. Models compared include: Light Gradient Boosting Machine (LGBM), TabNet, Variational Autoencoder (VAE), and Isolation Forest (IF). The “ALL” label refers to models trained on data from all age groups, while the “Specific” label refers to models trained only on the corresponding age group. Age groups are: 40–49 years (40s), 50–59 years (50s), 60–69 years (60s), 70–79 years (70s), and ≥ 80 years (80s and older). In the heatmap, green indicates AUC, and orange-based colors indicate sensitivity; darker shades represent higher values for the respective metric

**Fig. 3:**
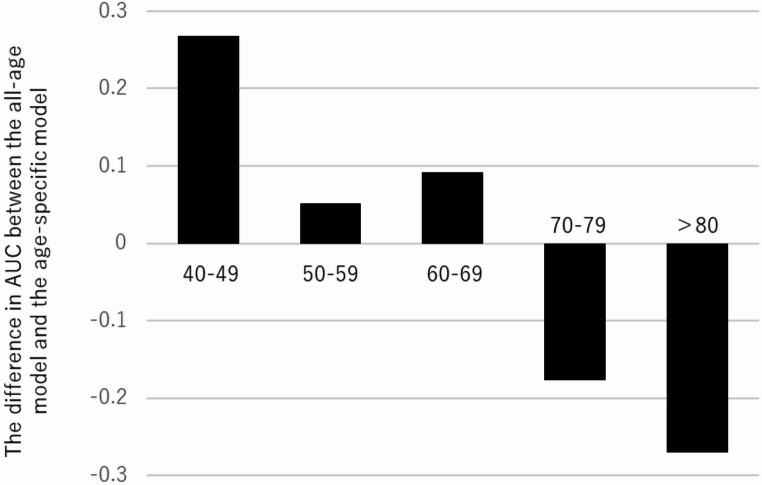
Difference in AUC between the LGBM all-age model and the age-specific models in external validation. A positive value indicates that the all-age model performed better, while a negative value indicates that the age-specific model performed better. The age group classifications are as follows: 40–49: 40s, 50–59: 50s, 60–69: 60s, 70–79: 70s, 80 and above: 80s and older

Figure 2 presents the results of the external validation conducted using health check-up data from Hakui City, comparing the predictive performance of the all-age model with that of the age-specific models. For participants in their 40s, 50s, and 60s, the all-age LGBM model demonstrated the highest AUC. However, for those in their 70s and 80s, the age-specific models outperformed the all-age model in terms of the AUC. Figure 3 shows the difference in AUC between the all-age and age-specific LGBM models.

Although models, such as TabNet, VAE, and IF, generally show high sensitivity, many have lower AUC and specificities. Specifically, the age-specific models (TabNet for those in their 40s and 50s, and VAE for those in their 60s and 80s) showed high specificity but exhibited lower sensitivity and AUC. Overall, a tradeoff between sensitivity and specificity was observed.

Figure 3 illustrates the differences in AUC between the all-age and age-specific LGBM models in the external validation. A positive value indicates superior performance of the all-age model compared with the corresponding age-specific model. The AUC was higher for the all-age model in participants in their 40s, 50s, and 60s, whereas the age-specific models achieved higher AUCs in participants in their 70s and those aged ≥ 80 years.

## DISCUSSION

This study had three distinct strengths. The first was the development of a general model for all age groups and age-specific models, demonstrating optimized DM prediction models tailored to each age group. Notably, in the external validation, the AUC comparison revealed that the all-age LGBM model performed better for individuals within their 40s to 60s, whereas the age-specific LGBM model demonstrated superior performance for those aged 70 years and older. Second, we proposed a novel DM prediction model that incorporates not only conventional ML methods, but also deep learning and an anomaly detection approach. This approach allowed us to clarify the impact of data imbalance and sample size variation on the model’s performance. Finally, we re-evaluated the generalizability of the model across different populations, thus enhancing its reliability.

In a review by Nomura et al., the AUC for models predicting new-onset DM using ML ranged from 0.71 to 0.872 [6–8, 26–31]. Lai et al.., as an example, developed a five-year DM prediction model based on data from 13,309 Canadian individuals aged 18–90. In this dataset, the incidence of DM within five years was 20.9%, and the issue of imbalanced data was addressed using a class-weighting method. Their gradient boosting model achieved an AUC of 84.7%, a sensitivity of 71.6%, and a specificity of 83.7% [29].

Similarly, Ravaut et al. predicted the onset of type 2 diabetes DM (T2DM) within five years using a large dataset of 2,137,343 individuals. In their study, 1,967 individuals developed T2DM within five years, accounting for 0.092% of the total cohort. They applied the synthetic minority oversampling technique, and their gradient boosting model achieved an AUC of up to 80.26%, with a sensitivity of 71.6% [31]. These findings suggest the importance of considering not only the total number of cases, but also the data imbalance between the target disease group and the rest of the population.

However, in previous studies on DM prediction models, sensitivity typically ranged from 42.2 to 71.6%; low sensitivity is a notable issue [6]. One of the main objectives of specific health checkups in Japan is the prevention and early detection of lifestyle-related diseases, such as DM. Therefore, highly sensitive models are desirable for screening. Currently, few reports exist on DM prediction algorithms that exhibit both high AUC and sensitivity. In our developed models, using a general model for all age groups or age-specific models, we demonstrated that the sensitivity and AUC outperformed previous studies in every age group.

In particular, the LGBM model showed high predictive accuracy across multiple age groups. The LGBM model employs a gradient-boosting ML framework based on decision trees. It is generally considered to have high predictive accuracy, as it tends to form more complex decision trees than other boosting ML algorithms, leading to improved prediction performance. Additionally, the LGBM model can handle missing data and provides feature importance metrics [15]. However, there was a risk of overfitting; therefore, we conducted hyperparameter tuning and a 50-fold cross-validation to ensure model stability.

TabNet is a deep-learning model for tabular data. While it is expected to perform as well as the LGBM model on tabular data, it is designed for large and complex datasets; for smaller datasets (fewer than several thousand records), the risk of overfitting or underfitting increases [16]. Specifically, in the 40s subgroup, both internal and external validations across all age groups had datasets with fewer than several thousand records, which may have decreased predictive accuracy.

Furthermore, data imbalance poses a significant challenge not only in terms of sample size but also in terms of data distribution. Generally, during population health checkups, normal data overwhelmingly outnumber abnormal or disease-related data, causing models to learn disproportionately from the majority class. Common methods for addressing imbalanced data include under- and oversampling, which are widely used in the development of disease prediction models.

Another method of addressing imbalanced data is to treat it as an anomaly detection problem, rather than a classification problem. Anomaly detection is a data mining technique used to identify data points that behave differently from the majority of accumulated data. The IF model uses a decision-tree-based anomaly-detection method [17], whereas the VAE model is neural-network-based and uses reconstruction errors for anomaly detection [18]. The IF model is particularly effective in detecting minority class data, whereas the VAE model can handle complex data structures and nonlinear data. In our study, the IF model showed the highest AUC and sensitivity in the 40s age group for internal validation, proving that it is particularly effective for imbalanced data.

This study had several limitations. First, the external validation was limited to data from a single municipality with a sample size of approximately 15,000 individuals. Therefore, further validation across municipalities and diverse populations is required. Evaluating the generalizability of the model across different regions and cultural backgrounds is a crucial task for future research. Second, we did not develop prediction models for individuals aged < 40 years. Given the increasing incidence of DM in younger populations, it is essential to develop models that account for the risk of DM onset in younger age groups to inform future interventions. Additionally, there is a risk that cases of type 1 or slowly progressive type 1 diabetes may be misclassified as type 2 DM. Incorporating mechanisms to accurately distinguish between these different types of diabetes is necessary to further improve prediction accuracy. Third, we did not conduct a feature importance analysis stratified by age group. Although models were developed separately for each age group, differences in key predictors remain unknown. Future work should incorporate age-specific analyses (e.g., SHAP) to clarify these differences [32 Finally, participant numbers and diabetes prevalence varied greatly by age group, which may have affected performance. This imbalance, despite mitigation with stratified sampling and undersampling, should be considered when interpreting age-specific results.

In conclusion, we developed general and age-specific models for all age groups, demonstrating the effectiveness of diabetes prediction models optimized for each age group. Moreover, we propose novel prediction models that incorporate deep learning and anomaly detection in addition to traditional ML methods, with a focus on disease prevalence. Future challenges include conducting external validation on broader populations, developing prediction models for individuals under 40 years of age, and implementing mechanisms to distinguish between different types of diabetes. Addressing these challenges will enable the development of more widely applicable models that can be used in clinical practice. Therefore, the development of diagnostic prediction algorithms is becoming increasingly widespread. It is crucial to focus on the prevalence of diseases across different age groups and the quality of databases used to develop high-performance prediction models. In future prospective studies, we plan to assess the effectiveness of active health guidance using this model to prevent the onset of diabetes.

## Supplementary Information

Supplementary Material 1

Supplementary Material 2

## Data Availability

De-identified participant data will be shared on a request basis. However, the data used in this study are restricted by the Kanazawa University IRB. Therefore, data will be made available upon reasonable request to Dr. Shigehiro Karashima, with permission from Kanazawa University, Kanazawa City Association, and Hakui City.

## References

[1] GBD, Diabetes collaborators. global, regional, and National burden of diabetes from 1990 to 2021, with projections of prevalence to 2050: A systematic analysis for the global burden of disease study 2021. Lancet. **402**, 2021–2023 (2023) 10.1016/S0140-6736(23)01301-6 PMC10364581 37356446

[2] P. Saeedi, I. Petersohn, P. Salpea, B. Malanda, S. Karuranga et al., (2019) Global and regional diabetes prevalence estimates for 2019 and projections for 2030 and 2045: Results from the International Diabetes Federation Diabetes Atlas, 9th edition. *Diabetes Res Clin Pract* (ninth edn.) 157: 107843 10.1016/j.diabres.2019.107843 31518657

[3] M. Lind, H. Imberg, R.L. Coleman, O. Nerman, R.R. Holman, Effects of early intensive glucose Lowering on the incidence of cardiovascular events in patients with newly diagnosed type 2 diabetes: an observational study in 98,658 patients in the Swedish National diabetes register. Diabetes Care. **44**, 2231–2237 (2021) 34244332 10.2337/dc20-2439 PMC8740943

[4] A. Gopalan et al., Behavioral interventions for diabetes self-management: A systematic review and meta-analysis. Diabetes Med. **35**, 1655–1662 (2018)

[5] K. Dulyapach, P. Ngamchaliew, P. Vichitkunakorn, P. Sornsenee, K. Choomalee, Trends in diabetes prevalence and associated risk factors in a middle-income country: A longitudinal study using thailand’s National surveys. Int. J. Public. Health. **67**, 1605039 (2022) 36518873 10.3389/ijph.2022.1605039 PMC9742202

[6] A. Nomura, M. Noguchi, M. Kometani, K. Furukawa, T. Yoneda, Artificial intelligence in current diabetes management and prediction. Curr. Diab Rep. **21**, 61 (2021) 34902070 10.1007/s11892-021-01423-2 PMC8668843

[7] Q. Zou, K. Qu, Y. Luo, D. Yin, Y. Ju et al., Predicting diabetes mellitus with machine learning techniques. Front. Genet. **9**, 515 (2018) 30459809 10.3389/fgene.2018.00515 PMC6232260

[8] L. Zhang, Y. Wang, M. Niu, C. Wang, Z. Wang, Machine learning for characterizing risk of type 2 diabetes mellitus in a rural Chinese population: the Henan rural cohort study. Sci. Rep. **10**, 4406 (2020) 32157171 10.1038/s41598-020-61123-x PMC7064542

[9] S.G. Choi, M. Oh, D.H. Park, B. Lee, Y.H. Lee et al., Comparisons of the prediction models for undiagnosed diabetes between machine learning versus traditional statistical methods. Sci. Rep. **13**, 13101 (2023) 37567907 10.1038/s41598-023-40170-0 PMC10421881

[10] L. Fregoso-Aparicio, J. Noguez, L. Montesinos, J.A. García-García, Machine learning and deep learning predictive models for type 2 diabetes: A systematic review. Diabetol. Metab. Syndr. **13**, 148 (2021) 34930452 10.1186/s13098-021-00767-9 PMC8686642

[11] D.P. Kingma, M. Welling, Auto-Encoding Variational Bayes. In: Proceedings of the 2nd International Conference on Learning Representations (ICLR). 2014

[12] F.T. Liu, K.M. Ting, Z.H. Zhou, (2008) Isolation forest. In: Eighth IEEE International Conference on Data Mining: 413–422

[13] J.L. Leevy, T.M. Khoshgoftaar, R.A. Bauder, N. Seliya, A survey on addressing high-class imbalance in big data. J. Big Data. **5**, 42 (2018)

[14] G. Ke, Q. Meng, T. Finley, T. Wang, W. Chen et al., LightGBM: A highly efficient gradient boosting decision tree. Adv. Neural Inf. Process. Syst. **30**, 3146–3154 (2017)

[15] S. Arik, T. Pfister, TabNet: attentive interpretable tabular learning. AAAI. **35**, 6679–6687 (2021)

[16] H. Tada, M.A. Kawashiri, K. Yasuda, M. Yamagishi, Associations between questionnaires on lifestyle and atherosclerotic cardiovascular disease in a Japanese general population: A cross-sectional study. PLOS ONE. **13**, e0208135 (2018) 30485359 10.1371/journal.pone.0208135 PMC6261639

[17] M. Kawakami, S. Karashima, K. Morita, H. Tada, H. Okada et al., Explainable machine learning for atrial fibrillation in the general population using a generalized additive model: A cross-sectional study. Circ. Rep. **4**, 73–82 (2022) 35178483 10.1253/circrep.CR-21-0151 PMC8811230

[18] R. Nambo, S. Karashima, R. Mizoguchi, S. Konishi, A. Hashimoto et al., Prediction and causal inference of cardiovascular and cerebrovascular diseases based on lifestyle questionnaires. Sci. Rep. **14**, 10492 (2024) 38714730 10.1038/s41598-024-61047-w PMC11076536

[19] G. Lemaître, F. Nogueira, C.K. Aridas, Imbalanced-learn: A python toolbox to tackle the curse of imbalanced datasets in machine learning. J. Mach. Learn. Res. **18**, 1–5 (2017)

[20] F. Pedregosa, G. Varoquaux, A. Gramfort, V. Michel, B. Thirion et al., Scikit-learn: machine learning in python. J. Mach. Learn. Res. **12**, 2825–2830 (2011)

[21] G.B.M.A. Light, accessed on fast, distributed, high performance gradient boosting framework based on decision tree algorithms. Microsoft Press. https://github.com/microsoft/LightGBM 2022 January 25

[22] PyTorch implementation of TabNet paper, DreamQuark-AI. https://github.com/dreamquark-ai/tabnet accessed on 2022 January 25

[23] M. Abadi, A. Agarwal, P. Barham, E. Brevdo, Z. Chen et al., TensorFlow: large-scale machine learning on heterogeneous distributed systems. OSDI (2016): 265–283

[24] D.J. Stekhoven, P. Bühlmann, MissForest: non-parametric missing value imputation for mixed-type data. Bioinformatics. **28**, 112–118 (2012) 22039212 10.1093/bioinformatics/btr597

[25] A.P.I. Tensorflow Keras, accessed on documents. https://www.tensorflow.org/api_docs/python/tf/keras 2025 August 24

[26] W.J. Youden, Index for rating diagnostic tests. Cancer. **3**, 32–35 (1950) 15405679 10.1002/1097-0142(1950)3:1<32::aid-cncr2820030106>3.0.co;2-3

[27] M. Carletti, M. Terzi, G.A. Susto, Interpretable anomaly detection with DIFFI: Depth-based isolation forest feature importance. arXiv preprint arXiv:2007.1111.

[28] B.G. Choi, S.W. Rha, S.W. Kim, J.H. Kang, J.Y. Park et al., Machine learning for the prediction of new-onset diabetes mellitus during 5-year follow-up in non-diabetic patients with cardiovascular risks. Yonsei Med. J. **60**, 191–199 (2019) 30666841 10.3349/ymj.2019.60.2.191 PMC6342710

[29] H. Lai, H. Huang, K. Keshavjee, A. Guergachi, X. Gao, Predictive models for diabetes mellitus using machine learning techniques. BMC Endocr. Disord. **19**, 101 (2019) 31615566 10.1186/s12902-019-0436-6 PMC6794897

[30] L. Kopitar, P. Kocbek, L. Cilar, A. Sheikh, G. Stiglic, Early detection of type 2 diabetes mellitus using machine learning-based prediction models. Sci. Rep. **10**, 11981 (2020) 32686721 10.1038/s41598-020-68771-z PMC7371679

[31] M. Ravaut, V. Harish, H. Sadeghi, K.K. Leung, M. Volkovs et al., Development and validation of a machine learning model using administrative health data to predict onset of type 2 diabetes. JAMA Netw. Open. **4**, e2111315 (2021) 34032855 10.1001/jamanetworkopen.2021.11315 PMC8150694

[32] M.L. Scott, L. Su-In, A unified approach to interpreting model predictions. NIPS. **17**, 4768–4777 (2017)

