# Supplementary material for: Development and validation of Age-Specific algorithms for diabetes prediction
Source: Endocrine. 2025 Sep 23;90(3):1253–62. doi: 10.1007/s12020-025-04428-z (PMC12708691; doi:10.1007/s12020-025-04428-z)
Supplement: Supplementary file 1 — Supplementary Material 1 [file 12020_2025_4428_MOESM1_ESM.docx]

**Supplementary Table S1. Attributes of health checkup data used for feature importance analysis**

**(A) Questionnaire-based variables**

| **Variable** | **Description** | **Response format** |
| --- | --- | --- |
| medication_antiHT | Use of antihypertensive drugs | Yes/No |
| medication_insulin | Use of insulin or oral hypoglycemic agents | Yes/No |
| medication_antiChol | Use of lipid-lowering drugs | Yes/No |
| p_stroke | History of stroke (ischemic or hemorrhagic) | Yes/No |
| p_CAD | History of coronary artery disease (angina or myocardial infarction) | Yes/No |
| p_CKD | History of chronic kidney disease or renal failure (including dialysis) | Yes/No |
| p_anemia | History of anemia | Yes/No |
| smoking | Current smoking habit | Yes/No |
| BW_increase | ≥10 kg weight gain since age 20 | Yes/No |
| regular_exercise | Regular exercise ≥30 min, ≥2 days/week, for ≥1 year | Yes/No |
| daily_exercise | Daily physical activity equivalent to walking ≥1 hour | Yes/No |
| walkspeed | Self-reported walking speed | Categorical |
| chewing | Chewing status during meals | Categorical |
| foodspeed | Usual eating speed | Categorical |
| dinner_time | Late-night dinner (within 2 h before bedtime, ≥3 times/week) | Yes/No |
| snack | Snacking or sweet beverage intake between meals | Yes/No |
| skip_breakfast | Skipping breakfast ≥3 times/week | Yes/No |
| alcohol | Drinking frequency | Categorical |
| drinking_amount | Alcohol intake per drinking day | Continuous (g ethanol/day) |
| sleep | Sufficient rest with sleep | Yes/No |
| life_habit | Willingness to improve lifestyle (exercise, diet) | Yes/No |
| want_to_learn | Intention to receive health guidance if available | Yes/No |

**(B) Clinical and laboratory variables**

| **Variable** | **Description** | **Unit** |
| --- | --- | --- |
| gender | Sex | Male/Female |
| age | Age | years |
| height | Height | cm |
| weight | Weight | kg |
| BMI | Body mass index | kg/m² |
| waist | Waist circumference | cm |
| SBP | Systolic blood pressure | mmHg |
| DBP | Diastolic blood pressure | mmHg |
| upro | Urinary protein | -, ±, 1+, 2+, 3+ |
| uglu | Urinary glucose | -, ±, 1+, 2+, 3+ |
| uOB | Urinary occult blood | -, ±, 1+, 2+, 3+ |
| blood_time | Sampling time (fasting/postprandial) | Categorical |
| ECGtest | Electrocardiogram (normal, mild abnormality, abnormal) | Categorical |
| m1–m9 | Minnesota code (ECG abnormalities) | Categorical |
| RBC | Red blood cell count | ×10⁴/µL |
| Hb | Hemoglobin | g/dL |
| Ht | Hematocrit | % |
| MCV | Mean corpuscular volume | fL |
| MCH | Mean corpuscular hemoglobin | pg |
| MCHC | Mean corpuscular hemoglobin concentration | % |
| WBC | White blood cell count | /µL |
| Plt | Platelet count | ×10⁴/µL |
| AST | Aspartate aminotransferase | U/L |
| ALT | Alanine aminotransferase | U/L |
| γGTP | Gamma-glutamyl transpeptidase | U/L |
| TC | Total cholesterol | mg/dL |
| HDLC | High-density lipoprotein cholesterol | mg/dL |
| LDLC | Low-density lipoprotein cholesterol | mg/dL |
| TG | Triglycerides | mg/dL |
| PG | Plasma glucose | mg/dL |
| HbA1c | Hemoglobin A1c (NGSP) | % |
| Cr | Serum creatinine | mg/dL |
| eGFR | Estimated glomerular filtration rate | mL/min/1.73 m² |
| UA | Serum uric acid | mg/dL |
| Physical exam (ex_) | Clinical findings (e.g., anemia, jaundice, arrhythmia, murmur, edema, neuropathy, cervical mass, others) | Presence/absence |

**Legend.** Supplementary Table S1 summarizes all questionnaire-based and clinical/laboratory variables included in the health checkup dataset used for model training and feature importance analysis (Supplemental Figure S1). Abbreviations shown in the feature importance rankings correspond directly to the variables listed in this table.
