# Supplementary material for: Development and validation of Age-Specific algorithms for diabetes prediction
Source: Endocrine. 2025 Sep 23;90(3):1253–62. doi: 10.1007/s12020-025-04428-z (PMC12708691; doi:10.1007/s12020-025-04428-z)
Supplement: Supplementary file 2 — Supplementary Material 2 [file 12020_2025_4428_MOESM2_ESM.docx]

Supplemental Figure 1


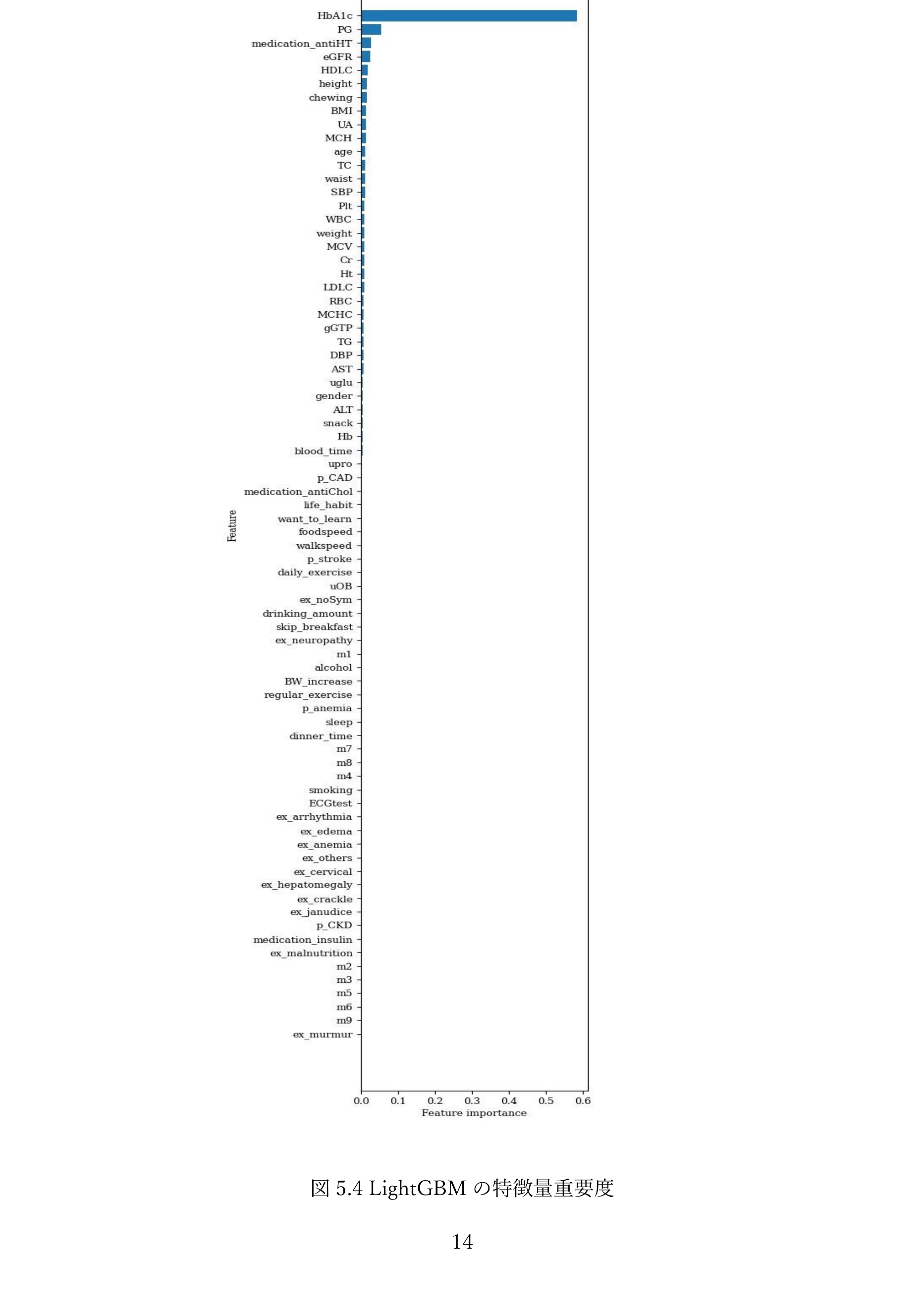


A.


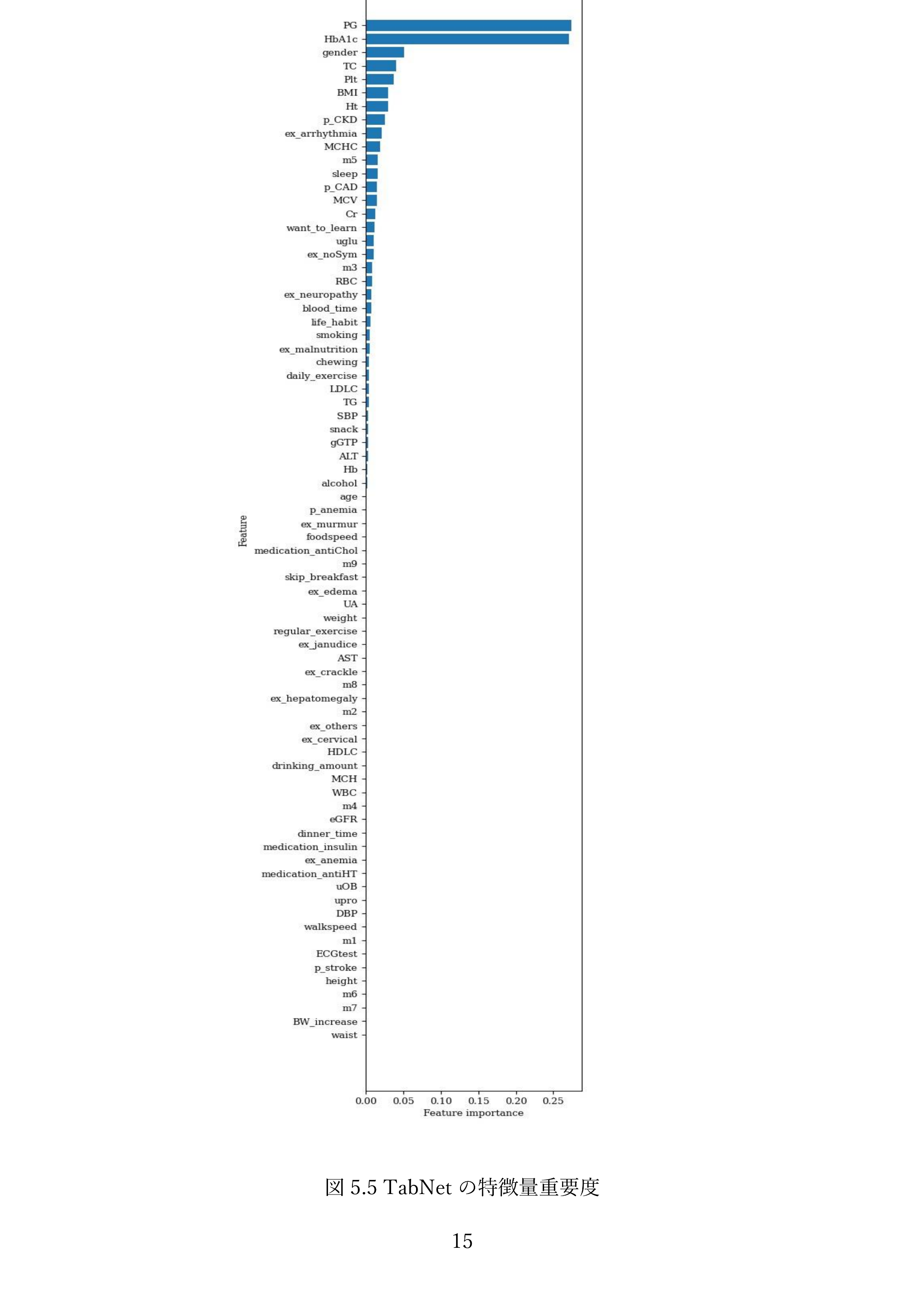


B.

**Figure Legend**

**Supplemental Figure S1. Feature importance ranking in LightGBM and TabNet models.**
Feature importance rankings derived from LightGBM (A) and TabNet (B) models are shown. Abbreviations on the vertical axis correspond to the clinical and questionnaire-based variables listed in Supplemental Table 1.
